# The effect of temperature on persistence of SARS-CoV-2 on common surfaces

**DOI:** 10.1186/s12985-020-01418-7

**Published:** 2020-10-07

**Authors:** Shane Riddell, Sarah Goldie, Andrew Hill, Debbie Eagles, Trevor W. Drew

**Affiliations:** Commonwealth Scientific and Industrial Research Organisation (CSIRO), Australian Centre for Disease Preparedness, Geelong, VIC Australia

**Keywords:** Environmental stability, SARS-CoV-2, COVID-19, Survivability

## Abstract

**Background:**

The rate at which COVID-19 has spread throughout the globe has been alarming. While the role of fomite transmission is not yet fully understood, precise data on the environmental stability of SARS-CoV-2 is required to determine the risks of fomite transmission from contaminated surfaces.

**Methods:**

This study measured the survival rates of infectious SARS-CoV-2, suspended in a standard ASTM E2197 matrix, on several common surface types. All experiments were carried out in the dark, to negate any effects of UV light. Inoculated surfaces were incubated at 20 °C, 30 °C and 40 °C and sampled at various time points.

**Results:**

Survival rates of SARS-CoV-2 were determined at different temperatures and D-values, Z-values and half-life were calculated. We obtained half lives of between 1.7 and 2.7 days at 20 °C, reducing to a few hours when temperature was elevated to 40 °C. With initial viral loads broadly equivalent to the highest titres excreted by infectious patients, viable virus was isolated for up to 28 days at 20 °C from common surfaces such as glass, stainless steel and both paper and polymer banknotes. Conversely, infectious virus survived less than 24 h at 40 °C on some surfaces.

**Conclusion:**

These findings demonstrate SARS-CoV-2 can remain infectious for significantly longer time periods than generally considered possible. These results could be used to inform improved risk mitigation procedures to prevent the fomite spread of COVID-19.

## Background

The World Health Organization (WHO) declared SARS-CoV-2 a pandemic on 11th March 2020 and as at the 7th August 2020, there have been over 18.8 million confirmed cases with more than 708,000 reported deaths from SARS-CoV-2 [1].

The transmission of SARS-CoV-2 appears to be primarily via aerosols [2–4] and recent studies have shown that SARS-CoV-2 is able to remain infectious in airborne particles for greater than 3 h [5, 6]. The role of fomites in the current pandemic is yet to be fully determined, although they have been suggested as a potential mode of transmission [7] also reflected by the strong focus on hand-washing by WHO and national control schemes. Broadly, viruses have been shown to be readily transferred between contaminated skin and a fomite surface [8], with high contact surfaces such as touchscreens on mobile phones, bank ATMs, airport check-in kiosks and supermarket self-serve kiosks all acting as fomites for the transmission of viruses [9]. Fomite transmission has previously been shown to be a highly efficient procedure, with transmission efficiencies of 33% for both fomite to hand and fingertip to mouth transfer for bacteria and phages [10]. With the high efficiency of fomite transfer, the persistence of SARS-CoV-2 on environmental surfaces is therefore a critical factor when considering the potential for fomite transmission for this virus. Currently, there are conflicting reports on the survivability of SARS-CoV-2, with data ranging from 3 to 14 days at room temperature for a single surface type, stainless steel [5, 11]. This study aims to provide environmental stability data for SARS-CoV-2 under controlled temperature and humidity conditions for a range of common surfaces.

## Methods

### Virus isolate

The SARS-CoV-2 isolate (*Betacoronavirus/Australia/SA01/2020*) used in this study was kindly supplied by the Peter Doherty Institute (Victoria, Australia) on behalf of South Australian Health (South Australia). The virus was passaged four times through Vero E6 cells (ATCC CRL-1586) in Dulbecco’s Modified Eagle Medium (DMEM) supplemented with Penicillin, Streptomycin, Fungizone and 10% fetal calf serum and pelleted via ultracentrifugation at 100,000×*g* for 90 min. The virus was resuspended in phosphate buffered saline (PBS) with 1% bovine serum albumin (BSA) and stored at − 80 °C. The virus stock was titrated on Vero E6 cells and the TCID_50_ was determined to be 4.97 × 10^7^/mL by the Spearman–Karber method [12, 13].

All work with infectious SARS-CoV-2 was conducted in the high containment laboratory (Biosafety level 4) at the Australian Centre for Disease Preparedness.

### Surfaces

Australian polymer bank notes, de-monetised paper bank notes and common surfaces including brushed stainless steel, glass, vinyl and cotton cloth were used as substrates in this study. Both polymer and paper banknotes were included in the study to gather information on the possible roles of note based currency in general for the potential for fomite transmission. Stainless steel is used in kitchen areas and public facilities and is the substrate used in some disinfectant testing standards [14, 15]. Glass was chosen due to its prevalence in public areas, including hospital waiting rooms, public transport windows and shopping centres, and high contact surfaces such as mobile phone screens, ATMs and self-serve check-out machines. Vinyl is a common substrate used in social settings, tables, flooring, grab handles on public transport, as well as mobile phone screen protector material. Cotton was chosen as a porous substrate, often found in clothing, bedding and household fabrics.

All surfaces were prepared by cutting into approx. 1–1.5 cm^2^ coupons, non-porous surfaces were disinfected prior to use by washing in a mild detergent (Beckman 555), rinsing in distilled water and then immersing in 80% v/v ethanol. Paper bank notes (in very good condition) were heated in a dry oven to 75 °C for 1 h to reduce bacterial/viral contamination. The 100% cotton cloth was steam sterilised prior to use.

Following preparation, all surfaces were placed into a petri dish and allowed to dry in a class II biological safety cabinet (BSCII) at room temperature and humidity prior to inoculation.

### Surface inoculation and sampling

Stock virus was diluted in a defined organic matrix, consisting of bovine serum albumin (BSA), mucin and tryptone, following international standard ASTM E2197 [15], designed to mimic the composition of body secretions. Briefly, 360 µL of virus stock was added to 160 µL of a solution consisting of 2.5 mg/mL BSA, 3.5 mg/mL tryptone and 0.8 mg/mL mucin. Ten microlitres of the resulting suspension (final concentration of 3.38 × 10^5^/10 µL) was inoculated onto the centre of the coupon and allowed to dry in a BSCII for 1 h. Once dry, the coupons were placed into a humidified climate chamber (Memmert HPP110) for specified time points. Samples were incubated in the dark to limit any effect light might have on viral decay. A single humidity set point (50% relative humidity) was maintained for each of three separate temperature experiments (20 °C, 30 °C, 40 °C). For the 20 °C and 30 °C temperature experiments, three replicates of each surface type were inoculated and sampled at the following time points; 1 h, 1 day, 3 days, 7 days, 14 days, 21 days and 28 days post inoculation. For the 40 °C experiment, triplicate samples were inoculated for the following time points; 1 h, 1 day, 2 days, 3 days, 4 days, and 7 days.

For non-porous surfaces, for each replicate, virus was eluted in 2 × 115 µL volumes of DMEM with repeated pipetting then titrated individually, in quadruplicate wells on a 96-well plate. For recovery from cotton cloth, inoculated swatches of the cloth were individually submersed in 500 µL DMEM and pipetted repeatedly for at least 1 min before 230 µL of the recovered eluent from each swatch was titrated separately, in quadruplicate. Suspensions of Vero E6 cells (3 × 10^5^/mL) were added to the wells and the plates were incubated for 3 days at 37 °C with 5% CO_2_. Wells were scored for the presence of cytopathic effect and titres calculated using the Spearman–Karber method.

### Statistical analysis

Data analysis (regression analysis) and graphical representations were performed using GraphPad Prism (version 5). Decimal reduction time (D value—time at which there was a one log/90% reduction in titre) was calculated using

$$D=\frac{t}{\left(\mathrm{log}{N}_{0}-\mathrm{log}{N}_{f}\right)}$$

Z-values (temperature change required to achieve a tenfold (i.e. 1 log_10_) change in the D value) was calculated by plotting log D values against temperature. Calculated using:

$$Z \, = \left( {t_{{2}} -t_{{1}} } \right)/\left( {{\log}D_{{1}} -{\log}D_{{2}} } \right)$$

The half-life of each surface was calculated using;

$${t}_{1/2}=\frac{{\mathit{log}}_{10}2}{k}$$

## Results

At 20 °C, infectious SARS-CoV-2 virus was still detectable after 28 days post inoculation, for all non-porous surfaces tested (glass, polymer note, stainless steel, vinyl and paper notes). The recovery of SARS-CoV-2 on porous material (cotton cloth) was reduced compared with most non-porous surfaces, with no infectious virus recovered past day 14 post inoculation. The majority of virus reduction on cotton occurred very soon after application of virus, suggesting an immediate adsorption effect. The calculated D values for surfaces at 20 °C ranged from 5.5 days for cotton to 9.1 days for paper notes and are shown in Table 1.

**Table 1:** Calculated D values (time taken to achieve a 90% reduction in titre) and half-life (time taken to achieve a 50% reduction in titre—in parentheses) for all surfaces at 20 °C, 30 °C and 40 °C

	D values (half-life)			Z value
	20 °C—days	30 °C—days	40 °C – hours	(°C)
Stainless steel	5.96 (1.80)	1.74 (12.6 h)	4.86 (1.5 h)	13.62
Polymer note	6.85 (2.06)	2.04 (14.7 h)	4.78 (1.4 h)	13.02
Paper note	9.13 (2.74)	4.32 (32.7 h)	5.39 (1.6 h)	12.43
Glass	6.32 (1.90)	1.45 (10.5 h)	6.55 (2.0 h)	14.65
Cotton	5.57 (1.68)	1.65 (11.0 h)	–	18.91
Vinyl	6.34 (1.91)	1.40 (10.1 h)	9.90 (3.0 h)	16.86

Calculated Z values (temperature shift required to alter D value by 1 log). No infectious virus was recovered for cotton cloth at 40 °C at 24 h, D values were not able to be calculated

At 30 °C, infectious virus was recoverable for 7 days from stainless steel, polymer notes and glass, and 3 days for vinyl and cotton cloth. For paper notes, infectious virus was detected for 21 days, although there was less than 1 log of virus recovered for both 14 day and 21 day time points. The D values for surfaces at 30 °C ranged from 1.4 days for vinyl to 4.9 days for paper notes (Table 1).

At 40 °C, virus recovery was significantly reduced compared to both 20 °C and 30 °C experiments. Infectious SARS-CoV-2 was not recovered past 24 h for cotton cloth and 48 h for all remaining surfaces tested. Greater than 4-log reduction (99.99% reduction from starting titre) was observed in less than 24 h at 40 °C on all surfaces. The D values for surfaces at 40 °C have been converted to hours as they were all less than 1 day, values ranged from 5 h for polymer notes to 10.5 h for vinyl (Table 1).

**Fig. 1:**
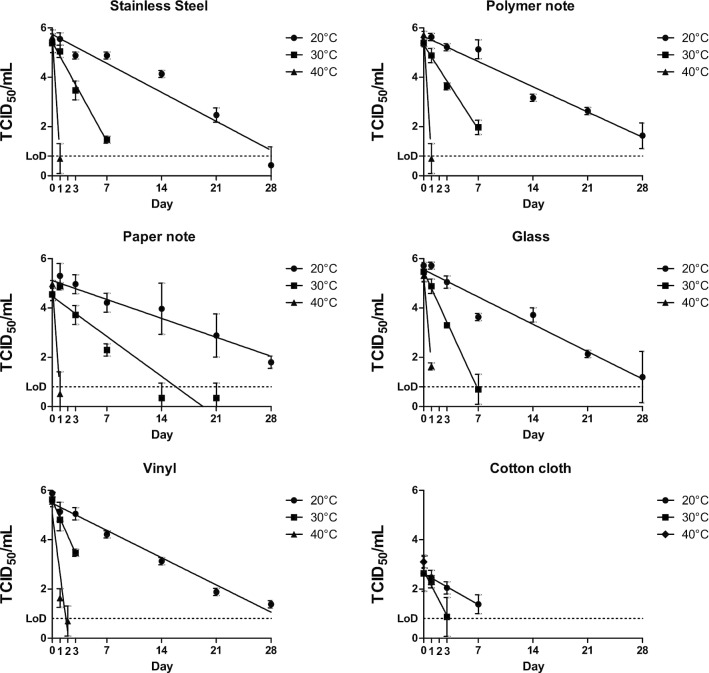
Recovery of infectious SARS-CoV-2 for all surfaces and temperatures over time, TCID_50_ data is plotted in log_10_ intervals. No infectious virus was recovered at 24 h at 40 °C for cotton cloth. LoD (limit of detection) is recorded as 0.8 Log_10_ TCID_50_

**Fig. 2:**
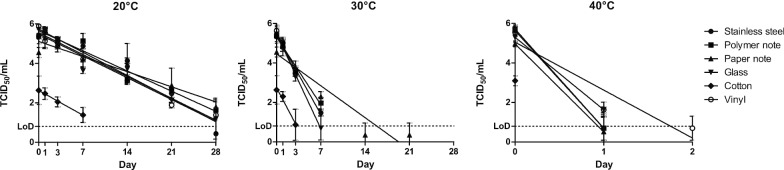
Grouping of each surface for individual temperatures. Trend lines for 20 °C show similar slopes, including for cotton cloth (although a reduced recovery was observed). A single well of virus was observed for paper banknotes in one out of three replicates for both 14 days and 21 days.

For each temperature and substrate material, the mean titre from three replicates of recovered virus was plotted against time, with standard deviations included. Linear regression was used to calculate a line of best fit. Plots showing virus survival on each substrate at the three temperatures investigated are shown in Fig. 1. Plots presenting this data grouping all substrates at each of the three temperatures are given in Fig. 2. Calculated D-value, Half Life and Z-value are presented in Table 1.

An additional table containing average titre and standard deviation for all substrates, time points and temperatures is available (See Additional file 1).

## Discussion

While the primary spread of SARS-CoV-2 appears to be via aerosols and respiratory droplets, fomites may also be an important contributor in transmission of the virus. Fomite transmission has been demonstrated as an important factor in the spread other coronaviruses such as porcine epidemic diarrhea virus [16], as well as being suspected for Middle East Respiratory Syndrome coronavirus [17], human coronavirus 229E and OC43 [18] and SARS-CoV-2 [7].

This study utilised a virus concentration of 4.97 × 10^7^/mL diluted into a standard solution which mimics body fluid composition (final concentration of 3.38 × 10^5^/10 µL inoculum), which equates to a cycle threshold (CT) value of 14.2, 14.0 and 14.8 for N gene, E gene and RdRp gene real time RT-PCR, respectively (unpublished data). Previous studies have shown some patients with high viral loads have recorded CT values of between 13 and 15 [19–21]. van Doremalen et al. [5] described their test material (10^5^ TCID_50_/mL) as having a CT of 20–22, which compared similarly to CTs reported from clinical patients [5, 22]. While the titre of virus utilised in this study is high it represents a plausible amount of virus that may be deposited on a surface.

The present study has demonstrated that in controlled conditions, SARS-CoV-2 at a starting viral load and in a fluid matrix equivalent to that typically excreted by infected patients, remains viable for at least 28 days when dried onto non-porous surfaces at 20 °C and 50% relative humidity. Research on the original SARS virus also showed recovery of infectious virus when dried on plastic for up to 28 days at room temperature and 40–50% RH [23]. Recent data published on SARS-CoV-2 survivability on hospital PPE observed viable virus up to 21 days post inoculation on both plastic and N95 mask material when held at room temperature [11], correlating with the data presented in this study. The persistence of SARS-CoV-2 on surfaces presented here and from Kasloff et al. [11] demonstrate significantly longer time points than previously published data for SARS-CoV-2 [5, 24]. These earlier studies reported recovery of infectious SARS-CoV-2 up to 3 days post inoculation and 4 days on non-porous surfaces, respectively. The titre of virus used in this study is at least 2 logs higher than used in the paper by van Doremalen et al. [5], which may account for the longer survivability. Work by Lai et al. has shown that stability of SARS virus was enhanced with higher concentrations [25]. Temperature and humidity are both critical factors in viral survivability with an increase in either being detrimental to virus survival [23, 26, 27]. Survivability on stainless steel coupons for transmissible gastroenteritis virus and murine hepatitis virus (both coronaviruses) was reduced with higher humidity’s and temperature [28] and survivability of Middle East Respiratory Syndrome coronavirus also followed a similar pattern [29]. The higher humidity of ~ 65% RH used by Chin et al. [24] may explain the shorter persistence of virus when compared to the data presented here.

SARS-CoV-2 has been shown to be rapidly inactivated under simulated sunlight [30, 31]. To remove any potential decay by light sources, inoculated coupons were held in the dark for the duration of the experiment.

Decimal reduction (D value; the timetaken to reduce the titre by 1 log) for SARS-CoV-2 at 20 °C and 50%RH ranged from 5.57 to 9.13 days (average 6.82) for all surfaces tested. This data is significantly longer than modelling predications performed by Guillier et al. [32]. The data presented here was performed under controlled conditions with fixed temperatures, relative humidity, suspension matrix and in the absence of light, which may explain the enhanced survivability observed in this study. The generation of Z values at different temperatures also allows for extrapolation of D values for each surface at other temperatures. The Z value represents the temperature change required to alter the D value by 1 log. For stainless steel, the D value was determined to be 6.48 days at 20 °C, and the Z value of 13.62 °C, therefore if the temperature was to drop by 13.62 °C from 20 °C (i.e. to 6.38 °C), then the D value would increase from 6.48 days to over 64 days. This data could therefore provide a reasonable explanation for the outbreaks of COVID-19 surrounding meat processing and cold storage facilities. The data also supports the findings of a recent publication on survival of SARS-CoV-2 on fresh and frozen food [33].

Stainless steel is a common surface for study of viral stability, and has been used to study the persistence on a number of viruses such as Ebola virus, hepatitis virus, Influenza A and Coronaviruses [28, 34–37]. This study demonstrates that SARS-CoV-2 is extremely stable on stainless steel surfaces at room temperature (> 28 days at 20 °C/50%RH) however, is less stable at elevated temperatures (7 days at 30 °C and < 48 h at 40 °C). Recovery of infectious virus on stainless steel has been observed for murine hepatitis virus and transmissible gastroenteritis virus for up to 28 days albeit at a lower humidity 20%RH [28]. Interestingly, the same study showed survivability at 20 °C and 50%RH was significantly less (4–5 days), further suggesting the humidity may play a significant role in virus survival.

The persistence of virus on both paper and polymer currency is of particular significance, considering the frequency of circulation and the potential for transfer of viable virus both between individuals and geographic locations. While other studies have shown that paper notes harbour more pathogens than polymer notes [38], this data demonstrates that SARS-CoV-2 persists on both paper notes and polymer notes to at least 28 days at 20 °C, albeit with a faster rate of inactivation on polymer notes. Data presented in this study for banknotes is significantly longer than reported for other respiratory viruses such as Influenza A (H3N2) which demonstrated survival up to 17 days at room temperature [39]. It is also noted that prior to SARS-Cov-2 being declared a pandemic, China had commenced decontamination of its paper based currency, suggesting concerns over transmission via paper banknotes existed at the time [40, 41]. The United States and South Korea have also quarantined bank notes as a result of the pandemic [42, 43]. It is important to note that after 28 days, infectious SARS-CoV-2 was also recovered from stainless steel, vinyl and glass, suggesting survivability on paper or polymer banknotes was not very different from the other non-porous surfaces studied.

The persistence on glass is an important finding, given that touchscreen devices such as mobile phones, bank ATMs, supermarket self-serve checkouts and airport check-in kiosks are high touch surfaces which may not be regularly cleaned and therefore pose a transmission risk of SARS-CoV-2. It has been demonstrated that mobile phones can harbour pathogens responsible for nosocomial transmission [44], and unlike hands, are not regularly cleaned [45]. The data presented in this study correlates well with previously published data for Influenza A (H1N1) which recovered infectious virus up to 22 days at 22 °C and 7 days at 35 °C [37]. The persistence of SARS-COV-2 on glass and vinyl (both common screen and screen protector materials, suggest that touchscreen devices may provide a potential source of transmission, and should regularly be disinfected especially in multi-user environments.

The persistence of both SARS and SARS-CoV-2 on cotton has been demonstrated to be significantly shorter than on non-porous surfaces [11, 25]. The data presented here also shows a significant decrease in titre of recovered virus after just 1 h drying at room temperature (20 °C) the amount of virus recovered from cotton swatches was approximately 99% less than for comparable virus recovery time points for non-porous material. To verify the reduced recovery on cotton, virus was eluted 5 min after depositing on the cotton, as well as 1 h, the titre of recovered virus after 5 min was similar to that of non-porous surfaces (data not shown) suggesting the process of drying down was a significant factor for cotton material but not from the non-porous surfaces. Recovery of virus from porous substrates is also likely to be reduced compared to non-porous substrates due to adherence of the virus to the fabric fibres. When the rate of viral inactivation is considered over time rather than the gross reduction from the initial inoculum there is a more subtle difference from the non-porous surfaces. The D values for cotton at 20 °C, when compared other materials, are not significantly different from other substrates (eg. 5.6 days for cotton vs. 6.3 days for vinyl), and the slopes of the line which suggests the decay rate of virus is similar across substrates. This study also demonstrates significantly longer survival times on cotton (7 days) than previous reported [11, 25]. This difference could be due to differences in the types of cotton material used, the current study used 100% cotton cloth, while previous studies used either a cotton gown or cotton t-shirt.

## Conclusions

The data presented in this study demonstrates that infectious SARS-CoV-2 can be recovered from non-porous surfaces for at least 28 days at ambient temperature and humidity (20 °C and 50% RH). Increasing the temperature while maintaining humidity drastically reduced the survivability of the virus to as little as 24 h at 40 °C. The persistence of SARS-CoV-2 demonstrated in this study is pertinent to the public health and transport sectors. This data should be considered in strategies designed to mitigate the risk of fomite transmission during the current pandemic response.

## Supplementary information

**Additional file 1**. Table of average titre and standard deviation for recovery of infectious SARS-CoV-2 for all substrates, time points and temperatures.

## Data Availability

All data generated or analysed during the study is included in the Additional file 1.

## Abbreviations

ASTM: American Society for Testing and Materials
ATM: Automatic teller machine
BSCII: Biological Safety Cabinet, Class 2
BSA: Bovine serum albumin
CO_2_: Carbon dioxide
CT: Cycle threshold
DMEM: Dulbecco’s Modified Eagle Medium
E gene: Envelope gene of SARS-CoV-2
N gene: Nucleocapsid gene of SARS-CoV-2
N95: Non-oil 95 mask
PBS: Phosphate buffered saline
RH: Relative humidity
RT-PCR: Reverse transcription polymerase chain reaction
RdRp: Ribonucleic acid dependant ribonucleic acid polymerase
SARS: Severe Acute Respiratory Syndrome
SARS-CoV-2: Severe Acute Respiratory Syndrome Coronavirus 2
TCID_50_: Tissue Culture Infectious Dose—Fifty
U/V: Ultraviolet
WHO: World Health Organisation
V/V: Volume per volume

## References

[1] Coronavirus disease (COVID-19) pandemic. https://www.who.int/emergencies/diseases/novel-coronavirus-2019.

[2] Stadnytskyi V, Bax CE, Bax A, Anfinrud P (2020). The airborne lifetime of small speech droplets and their potential importance in SARS-CoV-2 transmission. Proc Natl Acad Sci U S A.

[3] Morawska L, Milton DK (2020). It is time to address airborne transmission of COVID-19. Clin Infect Dis..

[4] Zhang R, Li Y, Zhang AL, Wang Y, Molina MJ (2020). Identifying airborne transmission as the dominant route for the spread of COVID-19. Proc Natl Acad Sci.

[5] van Doremalen N, Bushmaker T, Morris DH, Holbrook MG, Gamble A, Williamson BN (2020). Aerosol and surface stability of SARS-CoV-2 as compared with SARS-CoV-1. N Engl J Med.

[6] Smither SJ, Eastaugh LS, Findlay JS, Lever MS (2020). Experimental aerosol survival of SARS-CoV-2 in artificial saliva and tissue culture media at medium and high humidity. Emerg Microbes Infect.

[7] Cai J, Sun W, Huang J, Gamber M, Wu J, He G (2020). Indirect virus transmission in cluster of COVID-19 cases, Wenzhou, China, 2020. Emerg Infect Dis.

[8] Julian TR, Leckie JO, Boehm AB (2010). Virus transfer between fingerpads and fomites. J Appl Microbiol.

[9] Rolfe T, Nitti M. Touchscreens: the mosquito of the digital age. 2016. https://emist.com/infection-prevention-touchscreens-are-contaminated/.

[10] Rusin P, Maxwell S, Gerba C (2002). Comparative surface-to-hand and fingertip-to-mouth transfer efficiency of gram-positive bacteria, gram-negative bacteria, and phage. J Appl Microbiol.

[11] Kasloff SB, Strong JE, Funk D, Cutts TA. Stability of SARS-CoV-2 on critical personal protective equipment. medRxiv. 2020;2020.06.11.20128884. 10.1038/s41598-020-80098-3 PMC7806900 33441775

[12] Kärber G (1931). Beitrag zur kollektiven Behandlung pharmakologischer. Beitrag zur Kollekt Behandlung pharmakologischer Reihenversuche.

[13] Spearman C (1908). The method of “right and wrong cases” ('constant stimuli’) without Gauss’s formulae. Br J Psychol. 1908–1920.

[14] Sattar SA, Springthorpe VS, Adegbunrin O, Zafer AA, Busa M (2003). A disc-based quantitative carrier test method to assess the virucidal activity of chemical germicides. J Virol Methods.

[15] ASTM E2197. Standard quantitative disk carrier test method for determining bactericidal, virucidal, fungicidal, mycobactericidal, and sporicidal activities of chemicals. ASTM Int. 2015. https://www.astm.org/Standards/E2197.htm.

[16] Kim Y, Yang M, Goyal SM, Cheeran MCJ, Torremorell M (2017). Evaluation of biosecurity measures to prevent indirect transmission of porcine epidemic diarrhea virus. BMC Vet Res.

[17] Lee SS, Wong NS (2015). Probable transmission chains of Middle East respiratory syndrome coronavirus and the multiple generations of secondary infection in South Korea. Int J Infect Dis.

[18] Sizun J, Yu MWN, Talbot PJ (2000). Survival of human coronaviruses 229E and OC43 in suspension and after drying on surfaces: A possible source of hospital-acquired infections. J Hosp Infect.

[19] La Scola B, Le Bideau M, Andreani J, Hoang VT, Grimaldier C, Colson P (2020). Viral RNA load as determined by cell culture as a management tool for discharge of SARS-CoV-2 patients from infectious disease wards. Eur J Clin Microbiol Infect Dis.

[20] Kam K, Yung CF, Cui L, Tzer Pin Lin R, Mak TM, Maiwald M (2020). A well infant with coronavirus disease 2019 with high viral load. Clin Infect Dis.

[21] Huang Y, Chen S, Yang Z, Guan W, Liu D, Lin Z (2020). SARS-CoV-2 viral load in clinical samples from critically Ill patients. Am J Respir Crit Care Med.

[22] Zou L, Ruan F, Huang M, Liang L, Huang H, Hong Z (2020). SARS-CoV-2 viral load in upper respiratory specimens of infected patients. N Engl J Med.

[23] Chan KH, Peiris JSM, Lam SY, Poon LLM, Yuen KY, Seto WH (2011). The effects of temperature and relative humidity on the viability of the SARS coronavirus. Adv Virol..

[24] Chin AWH, Chu JTS, Perera MRA, Hui KPY, Yen H-L, Chan MCW (2020). Stability of SARS-CoV-2 in different environmental conditions. Lancet Microbe.

[25] Lai MYY, Cheng PKC, Lim WWL (2005). Survival of severe acute respiratory syndrome coronavirus. Clin Infect Dis.

[26] Aboubakr HA, Sharafeldin TA, Goyal SM (2020). Stability of SARS-CoV-2 and other coronaviruses in the environment and on common touch surfaces and the influence of climatic conditions: a review. Transbound Emerg Dis.

[27] Biryukov J, Boydston JA, Dunning RA, Yeager JJ, Wood S, Reese AL (2020). Increasing temperature and relative humidity accelerates inactivation of SARS-CoV-2 on surfaces. mSphere.

[28] Casanova LM, Jeon S, Rutala WA, Weber DJ, Sobsey MD (2010). Effects of air temperature and relative humidity on coronavirus survival on surfaces. Appl Environ Microbiol.

[29] van Doremalen N, Bushmaker T, Munster V (2013). Stability of Middle East respiratory syndrome coronavirus (MERS-CoV) under different environmental conditions. Eurosurveillance.

[30] Ratnesar-Shumate S, Williams G, Green B, Krause M, Holland B, Wood S (2020). Simulated sunlight rapidly inactivates SARS-CoV-2 on surfaces. J Infect Dis.

[31] Schuit M, Ratnesar-Shumate S, Yolitz J, Williams G, Weaver W, Green B (2020). Airborne SARS-CoV-2 is rapidly inactivated by simulated sunlight. J Infect Dis.

[32] Guillier L, Martin-Latil S, Chaix E, Thébault A, Pavio N, Le Poder S (2020). Modelling the inactivation of viruses from the Coronaviridae family in response to temperature and relative humidity in suspensions or surfaces. Appl Environ Microbiol.

[33] Fisher D, Reilly A, Kang A, Zheng E, Cook AR, Anderson DE (2020). Seeding of outbreaks of COVID-19 by contaminated fresh and frozen food. bioRxiv.

[34] Fischer R, Judson S, Miazgowicz K, Bushmaker T, Prescott J, Munster VJ (2015). Ebola virus stability on surfaces and in fluids in simulated outbreak environments. Emerg Infect Dis.

[35] Mbithi JN, Springthorpe VS, Sattar SA (1991). Effect of relative humidity and air temperature on survival of hepatitis A virus on environmental surfaces. Appl Environ Microbiol.

[36] Warnes SL, Little ZR, Keevil CW (2015). Human coronavirus 229E remains infectious on common touch surface materials. MBio.

[37] Dublineau A, Batéjat C, Pinon A, Burguière AM, Leclercq I, Manuguerra JC (2011). Persistence of the 2009 pandemic influenza a (H1N1) virus in water and on non-porous surface. PLoS ONE.

[38] Vriesekoop F, Russell C, Alvarez-Mayorga B, Aidoo K, Yuan Q, Scannell A (2010). Dirty money: an investigation into the hygiene status of some of the world’s currencies as obtained from food outlets. Foodborne Pathog Dis.

[39] Thomas Y, Vogel G, Wunderli W, Suter P, Witschi M, Koch D (2008). Survival of influenza virus on banknotes. Appl Environ Microbiol.

[40] Yeung J. China is disinfecting and destroying cash to contain the coronavirus. 2020. https://edition.cnn.com/2020/02/17/asia/china-is-disinfecting-cash-coronavirus-intl-hnk-scli/index.html.

[41] Wibawa T. China cleans bank notes in bid to limit coronavirus COVID-19 spread. ABC news (Australia). 2020. https://www.abc.net.au/news/2020-02-21/china-cleaning-money-limit-coronavirus-covid-19/11983364.

[42] Schroeder P, Irrera A. Fed quarantines U.S. dollars repatriated from Asia on coronavirus caution. 2020. https://www.reuters.com/article/us-health-coronavirus-fed-dollars/fed-quarantines-us-dollars-repatriated-from-asia-on-coronavirus-caution-idUSKBN20T1YT.

[43] Choi H. S.Korea’s central bank burns, quarantines cash in coronavirus precaution. 2020. https://uk.reuters.com/article/health-coronavirus-southkorea-money/s-koreas-central-bank-burns-quarantines-cash-in-coronavirus-precaution-idUKL4N2AZ1TL.

[44] Brady RRW, Wasson A, Stirling I, McAllister C, Damani NN (2006). Is your phone bugged? The incidence of bacteria known to cause nosocomial infection on healthcare workers’ mobile phones. J Hosp Infect.

[45] Olsen M, Campos M, Lohning A, Jones P, Legget J, Bannach-Brown A (2020). Mobile phones represent a pathway for microbial transmission: a scoping review. Travel Med Infect Dis.

